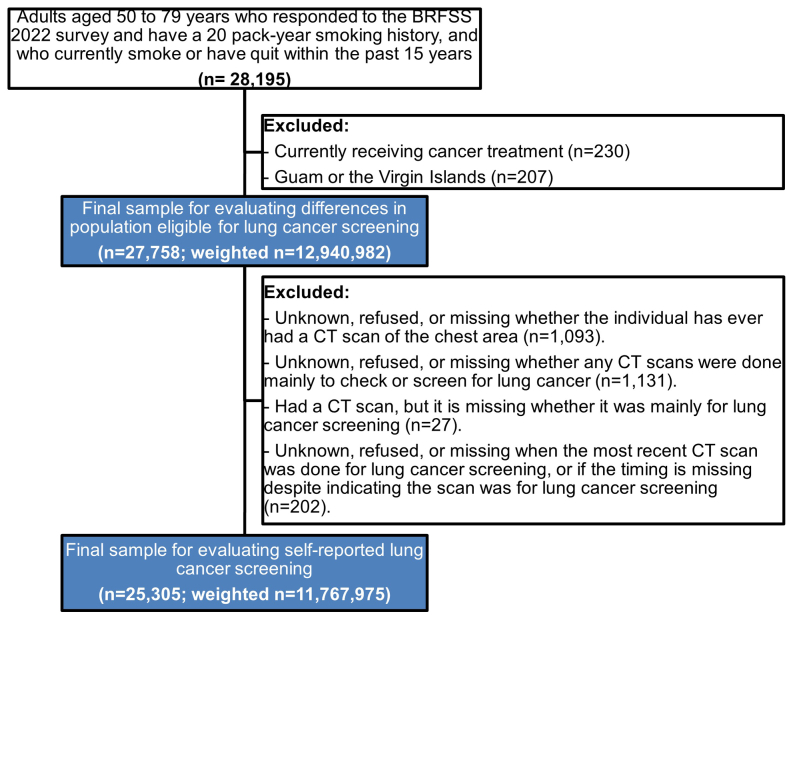# Lung Cancer Screening Eligibility, Uptake, and Adherence in Puerto Rico, 2022

**DOI:** 10.1016/j.jtocrr.2025.100852

**Published:** 2025-05-26

**Authors:** Maira A. Castañeda-Avila, Eduardo J. Santiago-Rodríguez, William Rodríguez-Cintrón, Coral Olazagasti, Efrén J. Flores, Estelamari Rodríguez, Ana I. Velázquez Mañana, Yomayra Otero-Domínguez, Eduardo R. Núñez

**Affiliations:** a Division of Epidemiology, Department of Population and Quantitative Health Sciences, University of Massachusetts Chan Medical School, Worcester, Massachusetts; b Division of Cancer Prevention, National Cancer Institute, Rockville, Maryland; c Division of Cancer Control and Population Sciences, National Cancer Institute, Rockville, Maryland; d Division of Pulmonary and Critical Care, San Juan VA Medical Center, San Juan, Puerto Rico; e Division of Medical Oncology, University of Miami Miller School of Medicine, Miami, Florida; f Department of Radiology, Massachusetts General Hospital, Boston, Massachusetts; g Division of Hematology/Oncology, University of California at San Francisco–School of Medicine, San Francisco, California; h Department of Medicine, University of Massachusetts Chan Medical School-Baystate, Springfield, Massachusetts; i Department of Healthcare Delivery and Population Sciences, University of Massachusetts Chan Medical School-Baystate, Springfield, Massachusetts

**Keywords:** Lung cancer screening, Puerto Rico, Cancer disparities, Hispanic or Latino health

## Abstract

**Importance:**

Lung cancer screening (LCS) with yearly low-dose computed tomography reduces lung cancer mortality, but uptake remains low. Puerto Rico, a U.S. territory, faces significant barriers to LCS implementation, but data on LCS eligibility and use are limited.

**Objective:**

This study aimed to estimate the number of individuals eligible for LCS in Puerto Rico and assess the prevalence of LCS use and up-to-date status compared with U.S. Hispanic and non-Hispanic populations.

**Design, Setting, and Participants:**

This cross-sectional study analyzed data from the 2022 Behavioral Risk Factor Surveillance System, a population-based telephone survey. Adults eligible for LCS per 2021 U.S. Preventive Services Task Force guidelines (aged 50–80 years, ≥20 pack-year smoking history, current or recent smokers) from Puerto Rico and the United States were included.

**Exposures:**

Participants were categorized into three groups: Puerto Rico residents, U.S. Hispanic, and U.S. non-Hispanic populations.

**Primary Outcomes and Measures:**

The primary outcomes and measures were self-reported receipt of initial LCS (ever had chest CT for screening) and being up to date with LCS (i.e., chest CT in the past year). Multivariable Poisson models estimated adjusted prevalence ratios for LCS outcomes.

**Results:**

After population weighting, 94,955 individuals were eligible for LCS in Puerto Rico, compared with 12.8 million in the U.S., representing 7.9% and 11.9% of their respective populations. The prevalence of self-reported LCS use was 28.4% in Puerto Rico, 27.6% among U.S. Hispanics, and 31.5% among U.S. non-Hispanics. Being up to date with LCS was lower among Puerto Rico residents (9.8%) than among U.S. Hispanics (17.3%) and non-Hispanics (18.1%). Multivariable models found Puerto Rico residents were less likely to be up to date with LCS than were U.S. non-Hispanics (adjusted prevalence ratios, 0.54; 95% CI 0.29–0.99).

**Conclusions and Relevance:**

Fewer than 10% of eligible individuals in Puerto Rico self-reported being up to date with LCS, indicating they are almost half as likely to self-report as eligible individuals in the United States, highlighting significant gaps in care. Implementing high-quality LCS in Puerto Rico is critical to reducing lung cancer mortality and providing equitable lung cancer care.

## Introduction

Lung cancer screening (LCS) with annual low-dose computed tomography (CT) has been reported to reduce lung cancer-related mortality by 20%.^1^^,^^2^ Nevertheless, only 5% to 18% of eligible individuals have received an initial LCS in the United States, with Hispanic individuals being less than half as likely to undergo screening as White individuals.^3^, ^4^, ^5^ Multilevel barriers limit implementation of LCS including patient (e.g., awareness, smoking-associated stigma, fear of cancer), provider (e.g., limited knowledge and time), and system-level factors (e.g., difficulty identifying eligible individuals, lack of administrative support).^6^ These barriers are likely to be compounded among Hispanic communities in the United States and its territories.^7^

Puerto Rico, a U.S. territory with a population of 3.2 million and high poverty rate (approximately 43% of the population lives below the U.S. federal poverty level), faces significant barriers to LCS. Previous studies have highlighted key challenges, including low patient and provider awareness, inadequate infrastructure for managing screen-detected findings, and uncertainty regarding insurance coverage.^8^, ^9^, ^10^ As a result, LCS is not routinely discussed in clinical settings. Achieving high-quality LCS in Puerto Rico has the potential to reduce lung cancer mortality and address longstanding disparities. Currently, the number of individuals in Puerto Rico who are eligible for LCS or have received it is not known.

Our study objectives were to (1) estimate the population eligible for LCS in Puerto Rico and (2) assess the current self-reported status of LCS in Puerto Rico compared with Hispanic and non-Hispanic U.S. populations.

## Methods

### Data Source

We analyzed data from the 2022 Behavioral Risk Factor Surveillance System (BRFSS), a telephone survey collecting health-related data from more than 400,000 U.S. residents annually, including those in all 50 states, the District of Columbia, and three U.S. territories, including Puerto Rico. BRFSS uses sampling and weighting methods to ensure data are representative across various demographic characteristics and geographic regions.

### Study Design and Population

This cross-sectional analysis included adults responding to the 2022 BRFSS survey who were eligible for LCS per the 2021 U.S. Preventive Services Task Force guidelines (aged 50–79 years, 20 pack-year smoking history, currently smoking, or quit within the past 15 years). We excluded respondents from Guam and the U.S. Virgin Islands and those currently receiving cancer treatment. The final sample included 25,305 respondents (weighted n = 11,767,975) (Supplementary Fig. 1).

### Measures

#### Outcomes

The outcomes of interest were self-reported: (1) initial receipt of LCS (ever had a CT Chest for lung cancer screening) and 2) being up to date with LCS (CT Chest for lung cancer screening in the last year).

#### Primary Exposure(s)

Participants were categorized into three groups: U.S. non-Hispanic, U.S. Hispanic, and Puerto Rico residents. Those residing in Puerto Rico were categorized as Puerto Rico residents, regardless of their race and ethnicity (99.2% self-reported Hispanic ethnicity).

#### Covariates

We a priori selected relevant covariables based on our prior research on factors related to cancer screening use.^11^, ^12^, ^13^ We included self-reported demographics (age, sex, race, ethnicity, marital status), socioeconomic factors (education, income, insurance), and health factors (general health, smoking status, comorbidities).

### Data Analysis

We summarized characteristics of participants eligible for LCS descriptive statistics and compared Puerto Rico, U.S. Hispanics, and U.S. non-Hispanics using chi-square tests. Poisson models estimated adjusted prevalence ratios (APRs) and 95% confidence intervals (CIs) for LCS status (never versus ever screened; never/not up to date versus up to date). Weights followed standardized protocols from the U.S. Centers for Disease Control and Prevention, accounting for complex survey design. The present study used publicly available, de-identified data and was deemed not human subjects research by Baystate’s institutional review board.

## Results

After population weighting, 94,955 individuals were eligible for LCS in Puerto Rico, compared with 12.8 million in the United States, representing 7.9% and 11.9% of their respective populations. Individuals in Puerto Rico who were eligible for LCS were more likely than those in the United States to be male, with lower educational attainment and income, and higher reliance on public insurance (Table 1).

**Table 1:** Sociodemographic and Clinical Characteristics of Adults Eligible for LCS by Ethnicity and Puerto Rican Residency (Unweighted n = 27,758; Weighted n = 12,940,982)

Participant Characteristics	U.S. Non-Hispanic (%)	U.S. Hispanic (%)	Puerto Rico (%)	*p* Value
**Total number of adults aged 50–79 y**^a^				
Unweighted, n	222,781	12,475	2809	
Weighted, n	95,519,812	12,803,883	1,204,814	
**Adults eligible for LCS**				
Unweighted, n	26,747	837	174	
Weighted, n	12,104,591	741,436	94,955	
**% of LCS eligible adults**	12.7	5.8	7.9	
**Age in y**				
50–59	34.3	42.0	36.2	0.042
60–69	43.2	40.5	43.5	
70–79	22.4	15.6	20.3	
**Male**	53.9	62.4	67.2	0.003
**Married/unmarried couple**	49.9	48.7	27.4	<0.001
**Multiracial race**				
White	83.5	82.6	59.4	<0.001
Black	9.2	7.1	40.0	
Other^b^	7.3	10.3	0.6	
**High school graduate or less**	52.9	62.4	65.5	0.001
**Income <$35,000**	44.7	52.1	91.9	<0.001
**Employed or self-employed**	35.4	40.5	22.3	0.040
**Urban counties**	89.1	96.4	-	<0.001
**Veteran status**	18.1	11.7	5.0	<0.001
**Health insurance**				
Public	63.0	60.7	79.9	0.041
Private	31.6	31.3	17.9	
Uninsured	5.5	8.0	2.2	
**Health care visit in the past year**	82.3	81.4	89.1	0.678
**Currently smoking tobacco cigarettes**	61.7	57.4	64.9	0.245
**Chronic obstructive pulmonary disease**	34.3	20.9	16.0	<0.001
**≥2 comorbid conditions**	61.2	50.1	60.0	<0.001

LCS, lung cancer screening.

a These are adults who participated in the Behavioral Risk Factor Surveillance System 2022 interview without any exclusions related to smoking status.

b Other includes multiracial, Asian, and American Indian/Native Hawaiian. Missing values: marital status (n = 124); multiracial race (n = 773); education (n = 66); income (n = 3954), employment (n = 114); metropolitan status (n = 180); urban/rural (n = 180); veteran (n = 42); insurance (n = 820); checkout (n = 250); personal provider (n = 191); could not afford to see doctor (n = 105); chronic obstructive pulmonary disease (n = 215).

**Figure 1:**
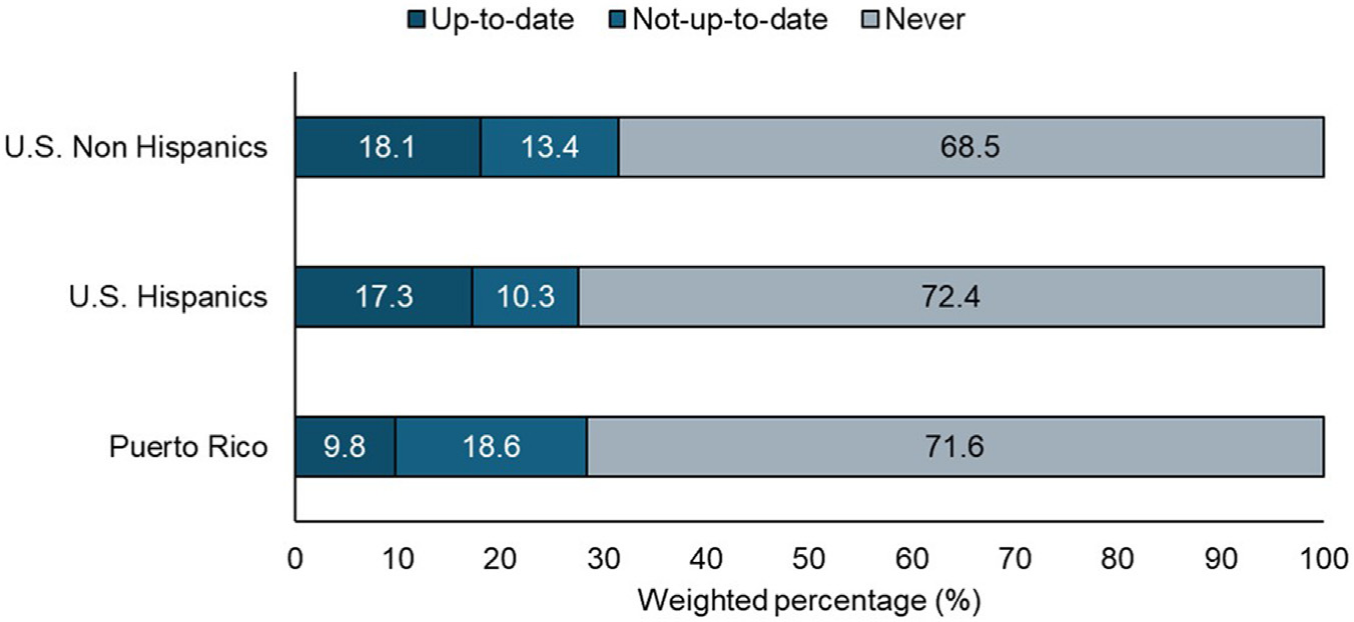
Lung cancer screening status among Puerto Rico, U.S. Hispanics, and U.S. non-Hispanic residents eligible for screening. Total sample: n = 25,305, weighted n = 11,767,975. U.S. non-Hispanic residents: n = 24,368, weighted n =10,989,484. U.S. Hispanic residents: n = 772, weighted n = 688,388. Puerto Rico residents: n = 165, weighted n = 90,153.

The prevalence of a self-reported initial LCS (i.e., ever had a CT Chest) was 28.4% in Puerto Rico, 27.6% among U.S. Hispanics, and 31.5% among U.S. non-Hispanics (Fig. 1). LCS ever use did not differ significantly between groups (Puerto Rico: APR, 0.89; 95% CI, 0.65–1.23; U.S. Hispanics: APR, 0.96; 95% CI, 0.79–1.18; U.S. non-Hispanic as reference).

The prevalence of being up to date with LCS was 9.8% in Puerto Rico, 17.3% among U.S. Hispanics, and 18.1% among U.S. non-Hispanics. Multivariable analyses found lower up-to-date LCS in Puerto Rico than in U.S. non-Hispanics (APR, 0.54; 95% CI, 0.29–0.99), whereas it was similar in U.S. Hispanics and U.S. non-Hispanics (APR, 1.06; 95% CI, 0.79–1.42) (Table 2).

**Table 2:** Association Between Individuals Living in Puerto Rico and Hispanics Living in the United States Compared With U.S. Non-Hispanics in Ever Having Had a Lung Cancer Screening and Being up to Date With Lung Cancer Screening

Participant Characteristics	Crude PRs (95% CI)	Adjusted PR (95% CI)^a^
**Ever vs. never (Ref.)**		
U.S. non-Hispanics	1.00	1.00
U.S. Hispanics	0.87 (0.70–1.08)	0.96 (0.79–1.18)
Puerto Rico	0.90 (0.66–1.23)	0.89 (0.65–1.23)
**Up to date vs. not up to date (Ref.)**		
U.S. non-Hispanics	1.00	1.00
U.S. Hispanics	0.95 (0.70–1.29)	1.06 (0.79–1.42)
Puerto Rico	0.54 (0.29–0.99)	0.54 (0.29–0.99)

n = 25,305, weighted n = 11,767,975.

U.S. non-Hispanic residents: n = 24,368, weighted n =10,989,484.

U.S. Hispanic residents: n = 772, weighted n = 688,388.

Puerto Rico residents: n = 165, weighted n = 90,153.

CI, confidence interval; PR, prevalence ratio; Ref., reference.

a Adjusted for age, sex, number of chronic conditions, insurance, and smoking status.

## Discussion

Almost 100,000 individuals in Puerto Rico were eligible for LCS in 2022, but only 9.8% were up to date, indicating a missed opportunity to decrease disparities in lung cancer outcomes through early detection. The proportion of individuals eligible for LCS is lower in Puerto Rico (8% versus 12% in the United States) owing to lower smoking prevalence, but the mortality rate for lung cancer in Puerto Rico is relatively higher, highlighting the importance of early detection.^10^

The study results reveal that although individuals in Puerto Rico self-report similar prevalence of ever having undergone LCS, they are nearly half as likely to be up to date with screening than are U.S. populations. Although 28% reported ever having a chest CT scan for screening, and 9.8% reported being up to date, these may be overestimates. For example, the American Lung Association reports that only 4.5% of eligible individuals in the United States have ever received an initial LCS, compared with 30% using BRFSS.^3^^,^^5^ The discrepancy between self-reported and claims-based estimates may be due to measurement bias (e.g., confusion among preventive screening, diagnostic, and surveillance testing), recall bias (e.g., inaccurate timing of screening), and selection bias (e.g., those who respond to BRFSS are more likely to engage in health care), which tend to overestimate actual LCS rates.^14^ Regardless of the actual utilization, LCS use in Puerto Rico is relatively lower, and disparities are expected to persist without intervention strategies.

Puerto Rico faces significant barriers to equitable implementation of LCS programs, including integrating LCS into routine practice, uncertainty about insurance coverage, and lack of accredited screening centers and a structure to manage screen-detected findings.^8^, ^9^, ^10^ Approximately 60% to 70% of Puerto Rican residents are covered by public insurance, which would cover LCS and downstream examinations and treatment for patients eligible for LCS.^10^ Nevertheless, awareness and access challenges persist, particularly among individuals with lower income. Providers may lack knowledge of LCS guidelines, and limited access to medical providers further complicates the necessary shared decision-making process. In addition, the ongoing brain drain (i.e., emigration of skilled professionals) in Puerto Rico, particularly in medical specialties, exacerbates these challenges by reducing the availability of providers to sustain LCS and manage screen-detected nodules.^15^ Although many individuals in Puerto Rico are covered by public insurance, Medicaid and Medicare funding is capped, and the federal matching rate is lower than in U.S. states, limiting investments in health care infrastructure. Indeed, a lack of dedicated LCS structures and processes (e.g., screening coordinators, tracking registries, multidisciplinary nodule boards, and a low number of American College of Radiology-Accredited imaging facilities) may contribute to the lower adherence to annual screening.

This study has limitations. BRFSS data are self-reported, which may lead to recall bias and overestimate actual LCS use. In addition, the U.S. Hispanic population is heterogeneous, making comparisons with Puerto Rico limited. Variations in survey response rates can influence selection bias; nevertheless, in 2022, Puerto Rico's BRFSS response rate was notably higher (58.5%) than the median response rate across all states and territories (45.1%), potentially reducing nonresponse bias in Puerto Rico.^16^ Despite these limitations, we believe this study is the first to evaluate LCS use in Puerto Rico. The analysis benefits from the most recent survey data that include cancer screening questions and a large, population-based sample for comparison. Future research could leverage administrative claims data or LCS registries to further explore screening patterns in Puerto Rico.

## Conclusion

Although approximately 100,000 individuals in Puerto Rico are eligible for LCS, fewer than 10% are up to date with screening, half as likely as U.S. individuals, highlighting a significant gap in care. In a resource-limited environment, primary prevention through tobacco treatment and secondary prevention through both initial uptake and annual adherence are essential for improving health outcomes. Engaging individuals in high-quality LCS must address barriers across the entire continuum, from awareness and identification of eligible patients to result-tracking and management of findings. Improving these processes is critical to reducing lung cancer mortality in Puerto Rico and can provide valuable insights for improving cancer care across Latin America.

## CRediT Authorship Contribution Statement

**Maira A. Castañeda-Avila**: Conceptualization, Methodology, Writing - original draft, review & editing.

**Eduardo J. Santiago-Rodríguez**: Conceptualization, Data curation, Formal analysis, Writing - original draft, review & editing.

**William Rodríguez-Cintrón**: Conceptualization, Investigation, Writing - review & editing.

**Coral Olazagasti**: Methodology, Investigation, Writing - review & editing.

**Efrén J. Flores**: Conceptualization, Methodology, Visualization, Writing - review & editing.

**Estelamari Rodríguez**: Investigation, Methodology, Writing - review & editing.

**Ana Velázquez Mañana**: Investigation, Methodology, Writing - review & editing.

**Yomayra Otero-Domínguez**: Investigation, Methodology, Writing - review & editing.

**Eduardo R. Núñez**: Conceptualization, Investigation, Methodology, Project administration, Writing - original draft, Writing - review & editing.

## Disclosure

The authors declare no conflict of interest.

## Figures and Tables

**Supplementary Figure 1:**